# Evaluation of the anthelmintic effectiveness of *Cinnamomum verum* bark extract in mice naturally infected with *Aspiculuris tetraptera*: *in vitro* and *in vivo*

**DOI:** 10.2478/helm-2025-0012

**Published:** 2025-09-30

**Authors:** M. Murshed, H. Alzaylaee, M. M. Mares, H. M. A. Aljawdah, S. Al-Quraishy

**Affiliations:** 1 Department of Zoology, College of Science, King Saud University, P.O. Box 2455, Riyadh 11451, Saudi Arabia; 2 Department of Biology, College of Science, Princess Nourah bint Abdulrahman University, P.O. Box 84428, Riyadh, 11451, Saudi Arabia

**Keywords:** *Cinnamomum verum* bark, Nematoda, bioassay, mortality, Albendazole

## Abstract

The health of the mice used in research experiments is critical to their performance in obtaining correct and accurate data. The current research was done to determine the anthelmintic action of *Cinnamomum verum* bark extracts (CVBE) in murine infection with *Aspiculuris tetraptera*. In vitro: The worms were divided into 6 groups: the 1^st^ control, 2^nd^ with 10 mg/mL of Albendazole as a reference drug, and the 3^rd^, 4^th^, 5^th^, and 6^th^ groups were treated with 25, 50, 100, and 200 mg/mL of CVBE, and the test was done at 15, 30, 60, and 120, minutes In vivo: Utilized was twenty-fi ve adult female (C57BL/6) murine, natural infections with *A. tetraptera*, were sectioned into fi ve treated, each containing 5 murine: 1^St^, was the non-treated (negative control), and 2^ed^ was regaled 100 μg/mL. 3^rd^ was regaled 200 μg/kg of CVBE for 5 days. 5^th^ was infection and regaled 10 mg/kg mebendazole for 3 days. The GC-MS analysis of phytochemicals in CVBE alcoholic extract disclosed the availability of 20 effi cacious bioactive compounds accountable for worm death. Mortality was measured a dose- and time-dependent effects starting in 20, 40, 80, 120, and 180 mins. The death rate reached (96 % and 100 %) through 120 and 180 min at engagement 200 and 400 mg/mL of CVE. While the non-treated groups lasted many times without death. This study demonstrated that CVBE was effective and had potent anthelminthic activity.

## Introduction

Gastrointestinal helminths are a big danger and cause signifi cant general health issues that can occasionally result in morbidity until death (Ranasinghe *et al*., 2023; Hassan *et al*., 2024). Although invisible to the human eye due to their small dimensions (250 μm to 12 mm in length and 15 to 35 μm in width), they pose a serious risk to human and animal health, which may result in growth retardation (Catani *et al*., 2023). Because helminth infections continuously contaminate the environment with their eggs and larvae, the persistent and asymptomatic character of the illness, especially in its early stages, maybe the cause of its neglect (Nalule *et al*., 2013). The soil-transmitted helminths, also known as intestinal nematodes, fi larial worms, schistosomes, and onchocerciasis worms are the most prevalent (Sayed *et al*., 2024). Contains several hosts for various phases. In addition, a major adaptive feature of a worm’s parasitism is its complex life cycle, which involves trophic transmission (Kasl *et al*., 2018). Certain soil-borne nematodes, including *Strongyloides* and hookworms, in their embryonic life cycle have two stages: the free-living stage, known as rhabditiform larvae, and the parasitic stage, known as fi lariform larvae, which may need a different host or habitat (Bruno *et al*., 2019). Gastrointestinal helminth and protozoa infections can affect a host’s capacity for survival and reproduction in two ways: directly, by causing pathological effects like tissue damage and blood loss; and indirectly, by lowering the host’s condition, making it more difficult for it to evade predators, and using up energy (Scantlebury *et al*., 2007).

First-stage larvae develop into third-stage larvae that bear to the colonic lumen and become adult larvae after a week of survival in the colon’s submucosa (Stepek *et al*., 2006). Mature females reside in the large intestine, where they spend forty to fifty days before ovulation. The stool pellets excreted at night are covered with a mucus layer by the eggs. It takes them 6 – 7 days at 24°C to become infectious, and they can survive for weeks outside of the host (Fox, 2015). Infections are usually asymptomatic. After the eaten eggs hatch, the larvae proceed to the middle colon, where they spend four to five days in crypts. They go on to the proximal colon after the host has been infected for around three weeks. The infection’s 10 – 12-day extended life cycle causes infestations to develop in slightly older mice; the worst infestation is predicted to occur 5 – 7 weeks after initial exposure (Omer *et al*., 2020). Significant variations in medication resistance and susceptibility between mouse strains of *A. tetraptera* are evident from observations of naturally occurring and experimental oxyuroid infections (Matté *et al*., 2023). Therefore, infection in lab mice cannot be avoided, and if treatment is not given, the infection will persist in the animals. Several researchers have demonstrated the influence of several parasites, including *A. tetraptera*, resulting in reduced immune-response, decreased hemoglobin, erythrocyte count, and serum albumin, which may negatively affect the results of experiments, even though infected animals cannot show clinical signs in immunocompetent experimental murine (Hernando *et al*., 2019; Hernando *et al*., 2025).

The use of deworming medications is a key component of helminth control efforts. The available synthetic medicines have side effects, and many parasites are resistant to medication, which means that the anthelmintic drugs now in use are not effective in controlling these organisms. (Oliveira *et al*., 2009). The growing interest in and awareness of the health benefits of natural resources, including herbs, spices, and fruits, has led to a heightened consumption of natural products as a safe and effective means of addressing illness and increasing overall health (Hegazy *et al*., 2023). Plants include various chemical compounds, such as polyphenols, flavonoids, and xanthones, many of which exhibit therapeutic properties or pharmacological advantages (Bagheri *et al*., 2024). In this context, pharmacological discovery has always come from nature, exploring potential plant-based antiparasitic medicines that are affordable, easily accessible, and promising substitutes. Thus, researching the anthelmintic properties of conventionally used plants could lead to more effective treatment alternatives (Ranasinghe *et al*., 2023). Plant secondary metabolites with biocidal activities, particularly essential oils (EOs), have been extensively studied during the past two decades (Fanelli *et al*., 2025).

*C. verum* bark is a plant species generally referred to as “sweet bay” or “bay laurel.” A fragrant angiosperm native laurel is a member of the camphor family (Lauraceae) (Paparella *et al*., 2022). In its native environment, this slow-growing plant can grow up to 60 feet (ca. 18 m) tall with a conical or pyramidal appearance. It is a member of the Lauraceae family of flowering plants (Paparella *et al*., 2022). What makes it unique is that it exhibits biological activity (Caputo *et al*., 2017). According to studies by Simić *et al*. (2004), Sırıken *et al*. (2018), Fernandez *et al*. (2020), Jemâa *et al*. (2012), and others, it has antifungal, antiviral, and antibacterial properties when combined with its extract and essential oils.

In this work, mice naturally infected with *Aspiculuris tetraptera* were used to test the anthelmintic efficiency of *C. verum* bark; this particular species of worm was also evaluated in vitro. It can therefore be applied to humans and other types of animals.

## Material and Method

### Preparing extract

A taxonomist from the Faculty of Science (Plant Biology), University of King Saud, certified the botanical identity of *C. verum* bark collected from the spice markets in Riyadh. 400 g of bark was desiccated at 40 °C, it was milled in the parasitology laboratory using a grinder (Moline M-06, Italy). It was subsequently immersed for 24 hours at 4 °C in 70 % methanol. Thereafter, the resulting extract was dried and concentrated utilizing a rotary vacuum evaporator (Yamato RE300, Japan) at reduced pressure and 40 °C. The crude extracts were produced and subsequently kept at -20 °C until utilized in an experiment (Manikandan *et al*., 2008).

### Extract Analysis by GCMS

Using a Trace GC-ISQ Quantum mass spectrometer system (Thermo Scientific, Austin, TX, USA), the extract of *C. verum* bark was investigated. A flow rate of 1 mL/min was employed. A GC-MS was equipped with a TG-5MS column (30 m, 0.25 mm ID, 0.25 mm film thickness), and about 1 μL of the material was injected into it. Helium gas was the carrier at a constant flow rate of 1 mL/min. The observed mass spectra fell between 50 and 500 m/z. The temperature was initially set for 10 min at 50 °C, increased to 250 °C at a rate of 5 °C/min, held at 300 °C/2 min, and then held at 350 °C/10 min. The phytochemical components were identified by comparing the recorded mass spectra of each part with the information stored in earlier libraries, such as NIST, Adams, Terpenoids, and Volatile Organic Compounds libraries. Each component’s relative percentage was determined using the retention time index, and the average peak area was then contrasted with the total of all peak areas.

### Collection of adult worms

A total of 70 mice were examined with comparable weights and ages. Then, isolating each mouse in a cage to collect excrement, we found that 25 of the mice had *A. tetraptera* pinworm infections. Mice were anesthetized with CO_2_ and dissected. Mice’s intestines were removed and cleaned using a 0.9 % NaCl saline solution. From the infected mice’s colon and cecum, adult worms were removed. The active adults were separated into small plastic plates and distributed in 9 cm Petri dishes in about 7 cc of sterile physiological saline at 23 °C using a stereomicroscope (PX51, Olympus Co., Tokyo, Japan). The *A. tetraptera* was identified following the taxonomic characteristics (Omer *et al*., 2020). The worms that were chosen were healthy, motile, and had a standard microscopic structure. The experiment was then initiated as soon as the worms were gathered.

### Adult Bioassay in vitro

*Aspiculus tetraptera* worms were tested in vitro using six groups: control negative and positive, and contained four concentrations of *C. verum* bark extract (25, 50, 100, and 200 mg/mL). Next, ten mature worms that were actively moving and at room temperature were added to each Petri plate. As positive and negative controls, a saline solution and 10 mg/mL of albendazole were made. 10 worms were placed in each petri dish. Three replicates for each group. Following the course of therapy, observations were conducted by timing the worms’ deaths at 15, 30, 60, and 120 minutes. When a surgical needle is used to contact the worms’ body sections and the petri dish is rocked, the worms are deemed dead if they remain still for 30 seconds.

Larvae or adult worms were tested to evaluate the effectiveness of the extract by motility in vitro. Sensitivity analyses were conducted using the subculture method (Alimi *et al*., 2021). The percentage of inhibition and motility in treated worms was calculated (Murthy & Chatterjee, 1999) using a total of 30 worms (in replicates of 10 worms) for the motility assay per test concentration to assess the vitality of the treated worms. Under a microscope, parasite motility was assessed during test periods of exposure to all levels. Mortality percentage was recorded at 15, 30, 60, and 120 min after treatments, and it is expressed as a percentage of control. (Stepek *et al*., 2006). The parasite mortality rate for the extract was calculated according to the following equation:

1 Mortality % = Control - Treatedsample / Control * s100%

### Experimental mice in vivo

Twenty-five adult C57BL/6 mice, weighing an average of 21 ± 2 g and naturally infected with *A. tetraptera* were utilized. The mice were between 11±2 weeks old. They were kept in pristine cages with 12-hour light-dark cycle and 20 °C ambient temperature as typical laboratory conditions. Animals were subjected to parasitological analyses using centrifugal sedimentation methods that were produced with salt water. The mice employed in the investigation were those that *A. tetraptera* naturally infected.

### Experimental design

The mice that were naturally injured with *A. tetraptera* were split into six treated groups: all groups contained 5 mice: The 1^st^ control (without treatment), the 2^nd^ received 10 μg/mL of Albendazole (Veterinary Agriculture Products Company, Amman, Jordan (V.A.P.C.O.)) as a reference drug for 3 days, and the 3^rd^ received 25 μg/mL, the 4^th^ received 50 μg/mL, the 5^th^ received 100 μg/mL, and the 6^th^ received 200 μg/mL. The *C. verum* bark extract was administered for five days.

### Treatment strategy in mice

Following treatment, the mice were split into two stages: The first step was gathering each group’s excrement for three days following therapy. the eighth day following the five-day course of therapy. 1g of excrement was removed from each group after each mouse was put in its cage. Following an analysis of the worm count, three mice from each treatment were anesthetized, and the gut of each treated mouse was opened and cleaned with saline solution. We gathered and characterized parasites using a stereomicroscope. The worm burden in each group was compared.

Similarly, the feces were gathered and checked, and the worms were tallied in the second stage, which took place a week following the first. Following this, the remaining mice were all anatomically together. A comparison was made of the worm burden through the two stages and among the groups.

### Statistical analysis

A completely randomized design (CRD) was employed for the bioassay analysis. In the adult bioassay of the *C. verum* bark extracts, a one-way analysis of variance (ANOVA) was performed using SAS 9.2 software (SAS, 2012). To compare mortality means, Duncan’s Multiple Range tests (P≤0.05) were employed. The presentation of all mortality data is as mean ± standard error (SE). The association between exposure time and death percentage was examined using a linear correlation.

## Ethical Approval

The study complies with Saudi Arabia’s ethical guidelines for utilizing animals in research (Ethics Agreement ID: KSU-SE-21-86).

## Results

### GC-MS results

**Fig. 1.**
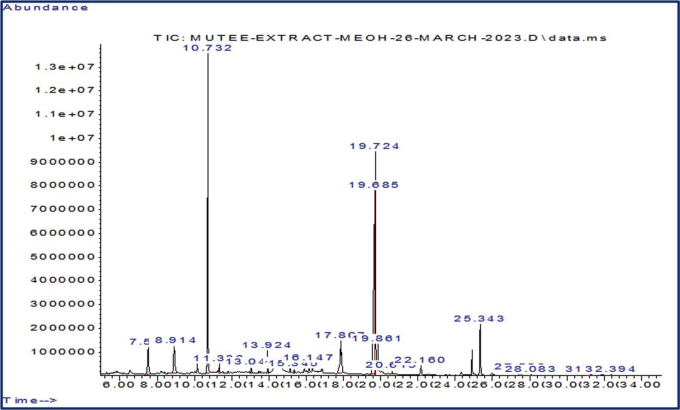
GCMS results of *Cinnamomum verum* bark extract.

The GC-MS analysis and qualitative phytochemical investigation of *C. verum* bark extracts showed 20 biologically active phytochemical compounds that were distributed throughout various peak areas and retention times (Fig. 1). The chemicals with the highest quantity found in CNB extract by GC-MS were 2-methyl benzofuran, pentadecanoic acid 14-methyl ester, cinnamon aldehyde, (E)-2-propenal-3-phenyl, methoxyphenyl, and hexadecanoic acid-2-methyl ester (Table 1). As can be seen, each chemical together with its pharmacological relevance in the fight against parasitic diseases was discussed.

**Tabe 1.** Identification of phytochemical compounds by GC-Mass in *Cinnamomum verum* bark extracts.

Retention time (min)	Bioactive phytochemicals	Molecular weight	Formula	Peak area %
7.53	4H-Pyran-4-one, 2,3-dihydro-3,5-dihydroxy-6-methyl-	144	C_6_H_8_O_4_	3.61
8.92	5-Hydroxymethylfurfural	126	C_6_H_6_O_3_	4.33
10.74	Eugenol	164	C_10_H_12_O_2_	25.89
11.34	Methyleugenol	178	C_11_H_14_O_2_	0.77
13.04	1,2,4-Cyclopentanetrione, 3-(2-pentenyl)-	180	C_10_H_12_O_3_	0.81
13.93	Methoxyeugenol	194	C_11_H_14_O_3_	1.30
15.14	2-Cyclohexen-1-one, 4-(3-hydroxybutyl)-3,5,5-trimethyl-	201	C_13_H_22_O_2_	0.33
15.35	Spathulenol	220	C_15_H_24_O	0.28
16.15	5,5,8a-Trimethyl-3,5,6,7,8,8a-hexahydro-2H-chromene	180	C_12_H_20_O	0.80
17.87	n-Hexadecanoic acid	256	C_16_H_32_O_2_	5.97
19.68	Linoleic acid	280	C_18_H_32_O_2_	32.84
19.72	Oleic Acid	282	C_18_H_34_O_2_	14.61
19.87	Octadecanoic acid	284	C_18_H_36_O_2_	1.72
20.62	Palmitoyl chloride	274	C_16_H_31_ClO	0.23
22.16	Linoleoyl chloride	298	C_18_H_31_ClO	1.35
25.34	Squalene	410	C_30_H_50_	4.52
27.52	γ-Tocopherol	416	C_28_H_48_O_2_	0.42
28.09	Stigmastan-3,5-diene	396	C_29_H_48_	0.11
31.30	Stigmasterol	412	C_29_H_48_O	0.34
32.39	Stigmast-7-en-3-ol	414	C_29_H_50_O	0.17

### In vitro

The effect of the extract from *C. verum* bark on *A. tetraptera* worms was studied in vitro using different concentrations of the extract (25, 50, 100, and 200 mg/mL), alongside the standard drug, 10 mg/mL albendazole, and distilled water as a control. Which was tested during 15, 30, 60, and 120 min to determine vitality and mortality rate. The results indicated that the mortality rate was low in worms exposed to reduced concentrations of extract 25 and 50 mg/mL, with death rates of 22.3 % and 38.7 %, while they had comparatively higher mortality rates at a concentration of 100 mg/mL, which amounted to 56.33 %, respectively. On the other hand, the worms that were exposed to high levels of extract concentration (200 mg/mL) and reference drug Albendazole (10 mg/mL) experienced very high death rates during 120 min, with death rates of 96 % and 100 %, respectively (Table 2).

**Table 2.** Means of mortality % (± SE) of *Cinnamomum verum* bark extract at various exposure duration (15, 30, 60, and 60 mins) against *Aspiculuris tetraptera*.

Concentration	Mortality (%)			
	15 min	30 min	60 min	120 min
Control	0.0 ± 0.0 ^c^	0 ± 0.0 ^c^	0 ± 0.0 ^c^	0 ± 0.0 ^c^
25 mg/mL	0.0 ± 0.0 ^c^	11± 3.5 ^abc^	4± 2.4 ^bc^	0 ± 0.0 ^c^
50 mg/mL	14.3± 2.7 ^abc^	35± 2.5 ^a^	23.3± 4.4 ^abc^	3.3± 2.7 ^bc^
100 mg/mL	26.7± 11.9 ^abc^	26.3± 22.4 ^ab^	45.7± 7.2 ^abc^	3.5± 2.5 ^bc^
200 mg/mL	40± 4.7 ^a^	33.3± 16.6 ^ab^	23.3± 11 ^abc^	3.3± 2.7 ^bc^
Albendazole 10 mg/mL	41 ± 3.2 ^a^	30±13.4 ^ab^	22.3± 11.9 ^abc^	4.3± 1.9 ^abc^

1 There is a significant difference (*p*< 0.05) between the means in columns or rows with distinct lowercase letters. indicates that there is no significant difference (p > 0.05) between those with identical lowercase letters, based on Duncan’s Multiple Range Test, conducted after ANOVA. The mean ± standard error is used to express each value.

**Fig. 2.**
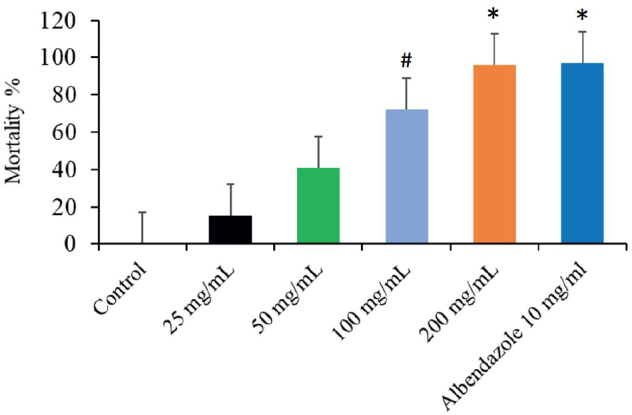
Impact of different dosages of *Cinnamomum verum* bark leaf extracts on *Aspiculuris tetraptera* mortality rates at 15, 30, 60, and 120 minutes (*Significant relative to the untreated group, p ≤0.01), # Significance of the untreated group statistically (p ≤0.05).

**Fig. 3.**
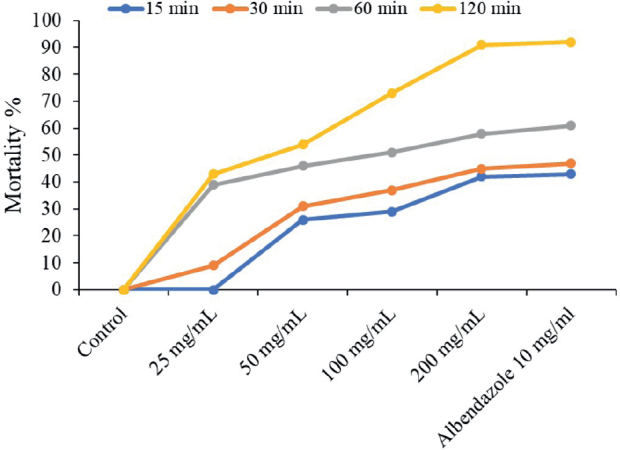
Mortality % of *Aspiculuris tetraptera* assayed with *Cinnamomum verum* bark extract (25, 50, 100, and 200 mg/mL) and the reference medication (10 mg/ml Albendazole), at various exposure durations (15, 30, 60, and 120 min).

The results indicated that the mortality percentage ranged from 13.3 % after 30 min at the 25 mg/mL concentration and 15 % after 60 min to 100 % after 120 min at the 200 mg/mL concentration. The highest average mortality % reached 41.3 % at 200 mg/mL concentration of CVBE after 15 min and 36.7 % at 10 mg/mL Albendazole after 30 min (Figs. 2, 3). The mortality percentage ranged from 7.7 % at a 50 mg/mL concentration to 33.3 % at 200 mg/mL after 30 min. The control group did not experience any mortality during the entire exposure time or at a 25 % concentration for 15 min. In the present investigation, the efficiency of extract concentrations increased with increasing exposure time (Fig. 2). The *A. tetraptera* worms generally have a higher mortality rate when they have longer times and high doses of the extract from *C. verum* bark.

**Fig. 4.**
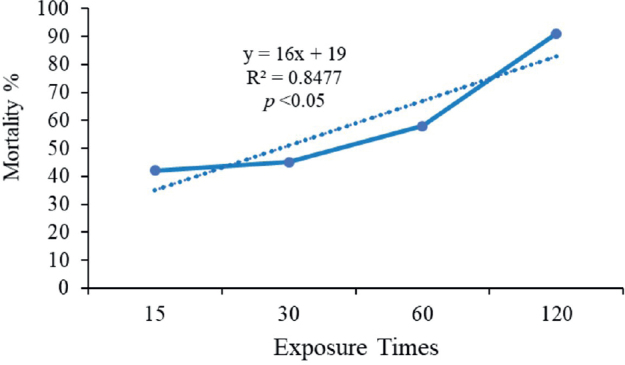
Mean mortality % of *Aspiculuris tetraptera* adults at 200 mg/mL concentration for various exposure times (hrs).

Significant differences (P < 0.05) were obtained in the *A. tetraptera* adult worm mortality percentage among bark extract concentrations (50, 100, and 200 mg/mL) compared to 25 mg/mL of CNB and the control. Overall, no significant differences were observed between 200 mg/mL concentrations with the albendazole 10 mg/mL (Fig. 4).

The mortality % was highly negatively correlated with exposure times [R^2^ = -0.98, P = .0001, (y = 16x + 19; R^2^ = 0.8477; p < 0.05)] at 200 mg/mL of CNB (Fig. 4). The overall results of the current study suggest that the extract of CNB may possess potential anthelmintic properties, which could potentially be employed in worm management.

### In vivo

**Fig. 5.**
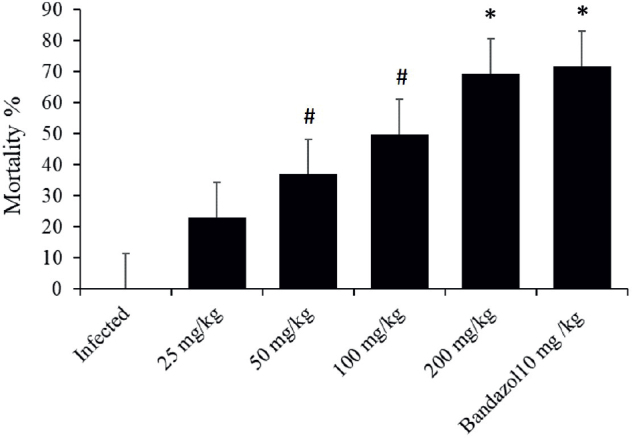
The general impacts of different dosages of *Cinnamomum verum* bark extracts (25,50, 100, 200 mg/ml), and reference drug (10 mg/ml Albendazole) on the mortality rate of A. tetraptera of 15 to 120 minutes. (*Significant relative to the untreated group, p ≤0.01), # Significance of the untreated group statistically (p ≤0.05).

**Fig. 6.**
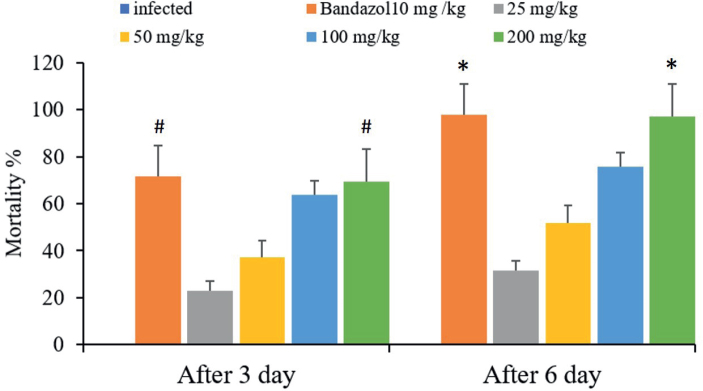
Principal effects of extracts from the bark of *Cinnamomum verum* at several dosages on the mortality rate of *Aspiculuris tetraptera* at 20, 40, 80, 120, and 180 minutes (*Significant relative to the untreated group, p ≤0.01). # Significance of the untreated group statistically (p ≤0.05).

The concentrations of the bark extract were evaluated to determine the optimal concentration that results in a high worm mortality rate. The concentration of 200 mg/kg showed the most lethal effects for worms (Fig. 5). Three days after giving the treatment, the murine was dissected. The results showed that the mortality rate of the worms counted from the murine’s intestines was 96 % at a concentration of 200 mL/kg of the extract and 89 % for the murine treated with a dose of 10 mL/kg of albendazole. Whereas the mortality rate of the worms taken from the gut of the treated mice that were dissected six days after giving the treatment was 100 % at a concentration of 200 mL/kg of the extract and 97 % for the mice treated with a dosage of 10 mL/kg of albendazole (Fig. 6). It is shown in Table 3 that mortality in the group treated with 200 mg/kg of CVBE and 10 mg/kg of albendazole decreased the count of eggs in the stool and even arrived at zero on the sixth day, as well as when dissected in the gut (Table 3). The concentration of 200 mL/kg demonstrated the most influence on worm mortality (Fig. 6).

**Table 3.** The average number of *A. tetraptera* worms obtained by fecal and intestinal centrifugation flotation per cage per mouse at necropsy in a strain of mice treated with *Cinnamomum verum* bark extract and the anthelmintic drug.

Groups	*n*	Average no. of worms after 3 days of treatment		Average no. of worms after 6 days of treatment	
		In feces	In intestinal	In feces	In intestinal
Infected	5	245±45	395±57	284±25	367±45
25 mg/kg	5	213±15	275±15	220±5	156±11
50 mg/kg	5	75±11	121±15	110±5	112±9
100 mg/kg	5	48±11	90±15	20±5	25±9
200 mg/kg	5	2±1.1	7±1	0	0
Albandazol 10 mg/kg	5	3±1	11±2	0	0

## Discussion

Plant products possess significant medicinal impacts for treating numerous contagious diseases (Beshbishy *et al*., 2019). This property makes medicinal plants an attractive choice as a source of new therapeutic compounds (Verdú *et al*., 2023). Despite its antiparasitic effect, researchers have not evaluated CNB against *A. tetraptera*. So, this study looks at how well CNB extract can kill *A. tetraptera* worms in lab tests and in living organisms. In this study, we noticed an increase in the death rate of the worm as exposure time progressed. Mortality ratios of 56.33 % – 100 % were noticed following treatment durations of 120 minutes for *A. tetraptera* worms with concentrations of 100 and 200 mg/mL of CNB extract and 96 % at 10 mL/mL albendazole, compared to the control (distilled water). The mortality rate was low for the low-concentration treatments (25 and 50 mg/mL). The results indicated that the CNB extract had a strong impact on *A. tetraptera* worms at all the concentrations tested in the lab (Mares *et al*., 2024). This study suggests that the CNB extract could be a potential alternative treatment for parasitic infections caused by *A. tetraptera*. We need further studies to explore the mechanism of action and evaluate the efficacy and safety of CNB extract in vivo.

The current results align with those reported by other researchers using various types of parasites. Sebai *et al*., (2022) discovered that 4 mg/mL of CNB essential oil exposed adult worms to 87.5 % immobility after 8 hours, exhibiting an inhibitory impact against *H. contortus* egg hatching with an inhibition value of 1.72 mg/mL. The plant’s yield *C. verum* bark essential oil, which contains D-limonene, linalool, and monoterpenes 1,8-cineol. Previous research has demonstrated the promising biological activity of plant linalool-rich extracts, including antibacterial, antiparasitic, and cytotoxic effects. Discovered that the most prevalent component in extracts from *Cinnamomum camphora* bark was linalool. Yang *et al*. (2014) reported that linalool showed effects on Schistosoma japonicum cercaria and snails in vitro. The *C. verum* bark extract demonstrated both larval effectiveness and acaricidal activity at various doses and times (Alimi *et al*., 2021).

The study revealed that the chemical composition of CNB essential oil works to inhibit both the antibacterial and antibiofilm activities against clinical Staphylococcus aureus strains (Merghni *et al*., 2016). In vivo, on the sixth day from the beginning of the experiment, all the rats were slaughtered, and the intestines, especially the caecum, were taken and opened in a Petri dish in the middle of saline solution. The worms in each group were counted and compared between all groups. We noticed that the number of worms obtained in the group injected with a concentration of 200 mg/mL CNB extract was deficient and almost nonexistent compared to the untreated infected group. Also, in the other groups dosed orally with concentrations of 100, 50, and 25 mg/kg, the percentage of worms obtained after examination under the microscope was low compared to the infected control group. Where he noticed that the effectiveness of the 200mg/kg concentration of the CNB extract was more effective at evicting worms than the albendazole treatment used commercially to eliminate worms. The results of this study are in line with numerous earlier investigations that confirmed the efficacy of CNB leaves in combating a range of parasites. After 7 days of treatment and a 79.2 % decrease in the overall number of worms, Sebai *et al*., 2022, discovered that the in vivo anthelmintic potential of CNB extract abolished the egg output of *Heligmosomoides polygyrus*. Furthermore, after a challenge infection, the amount of recovered schistosomulum from mouse skin was significantly decreased by the linalool found in the CNB plant. According to Yang *et al*. (2014), it reduced the worm load in infected animals. Linalool, which is essential for getting rid of worms in sick mice’s intestines, is abundant in the bark of the CNB plant (Sebai *et al*., 2022). Strong antibacterial, antimicrobial, and antioxidant properties have been reported for CNB (Koike *et al*., 2015). Traditionally, CNB has been used to treat gastrointestinal symptoms, such as eructation, epigastric bloating, impaired digestion, and flatulence (Kivçak *et al*., 2022).

The suitable anti-parasitic properties of *C. verum* bark were found in this study and others, suggesting that they could replace chemical medications in parasite control initiatives.

## Conclusion

This page lists the components of medicinal plants that are effective against gastrointestinal parasites both in vivo and in vitro. The results of these investigations indicate that herbal remedies hold great promise for creating new medications to treat parasitic illnesses, and the plant derivatives found in them provide useful structures for drug manufacturing and bioactivity optimization. Furthermore, the products of these plants provide useful compounds for the manufacture of pharmaceuticals. Plant medicament also hold great promise for the development of innovative therapies to treat parasite illnesses.

## References

[j_helm-2025-0012_ref_001] Alimi D., Hajri A., Jallouli S., Sebai H. (2021). *In vitro* acaricidal activity of essential oil and crude extracts of *Laurus nobilis*, (Lauraceae) grown in Tunisia, against arthropod ectoparasites of livestock and poultry: *Hyalomma scupense* and *Dermanyssus gallinae*. Vet Parasitol.

[j_helm-2025-0012_ref_002] Amirmohammadi M., Khajoenia S., Bahmani M., Rafieian-Kopaei M., Eftekhari Z., Qorbani M. (2014). *In vivo* evaluation of antiparasitic effects of *Artemisia abrotanum* and *Salvia officinalis* extracts on *Syphacia obvelata, Aspiculoris tetrapetra* and *Hymenolepis nana* parasites. Asian Pac J Trop Dis.

[j_helm-2025-0012_ref_003] Bagheri E., Shori A.B, Peng C.W., Baba A.S., Alzahrani A.J. (2024). Phytochemical analysis and medicinal properties of some selected traditional medicinal plants. Int J Agric Biosci.

[j_helm-2025-0012_ref_004] Beshbishy M., Batiha G. S., Adeyemi S., Yokoyama N., Igarashi I. (2019). Inhibitory effects of methanolic *Olea europaea* and acetonic *Acacia laeta* on growth of *Babesia* and *Theileria*. Asian Pac J Trop Med.

[j_helm-2025-0012_ref_005] Bruno R., Maresca M., Canaan S., Cavalier J.F., Mabrouk K., Boidin-Wichlacz C., Tasiemski A. (2019). Worms’ antimicrobial peptides. Mar Drugs.

[j_helm-2025-0012_ref_006] Caputo L., Nazzaro F., Souza L.F., Aliberti L., De Martino L., Fratianni F., De Feo V. (2017). *Laurus nobilis*: Composition of essential oil and its biological activities. Molecules.

[j_helm-2025-0012_ref_007] Catani L., Manachini B., Grassi E., Guidi L., Semprucci F. (2023). Essential oils as nematicides in plant protection - A review. Plants.

[j_helm-2025-0012_ref_008] Falcón-Ordaz J., Pulido-Flores G., Monks S. (2010). Una nueva especie de *Aspiculuris* (Nematoda: Heteroxynematidae), parásito de *Mus musculus* (Rodentia: Muridae), de Hidalgo, México [New species of *Aspiculuris* (Nematoda: Heteroxynematidae), parasite of *Mus musculus* (Rodentia: Muridae), from Hidalgo, Mexico]. Rev Mex Biodivers.

[j_helm-2025-0012_ref_009] Fanelli E., Vovlas A., D’addabbo T., De Luca F. (2025). Molecular mechanism of *Cinnamomum zeylanicum* and *Citrus aurantium* essential oils against the root-knot nematode, *Meloidogyne incognita*. Sci Rep.

[j_helm-2025-0012_ref_010] Fernandez C.M., Da Rosa M.F., Fernandez A.M., Bortoluc-ci W.C., Ferreira F.P., Linde G.A., Gazim Z.C. (2020). Essential oil and fractions isolated of Laurel to control adults and larvae of cattle ticks. Nat Prod Res.

[j_helm-2025-0012_ref_011] Gerwin P.M., Ricart Arbona R.J., Riedel E.R., Lepherd M.L., Henderson K.S., Lipman N.S. (2017). Evaluation of traditional and contemporary methods for detecting *Syphacia obvelata* and *Aspiculuris tetraptera* in laboratory mice. J Am Assoc Lab Anim Sci.

[j_helm-2025-0012_ref_012] Hassan N.F., El-Shemy A., El-Ezz N.A., Allam A.M., El-Shanawa-ny E.E. (2024). Intensity of gastrointestinal parasites and the associated risk factors, and sero-prevalence of hemonchosis among camels in Egypt. Int J Vet Sci.

[j_helm-2025-0012_ref_013] Hegazy S.A., Abd Elmawla S.M., Khorshed M.M., Salem F.A. (2023). Productive and immunological performance of small ruminants offered some medicinal plants as feed additives. Int J Vet Sci.

[j_helm-2025-0012_ref_014] Hernando G., Turani O., Bouzat C. (2019). Caenorhabditis elegans muscle Cys-loop receptors as novel targets of terpenoids with potential anthelmintic activity. PLoS Negl Trop Dis.

[j_helm-2025-0012_ref_015] Hernando G., Turani O., Rodriguez Araujo N., Pulido Carras-quero A., Bouzat C. (2025). Unraveling anthelmintic targets and mechanisms of action of trans-cinnamaldehyde from cinnamon essential oil. Sci Rep.

[j_helm-2025-0012_ref_016] Idris O.A., Wintola O.A., Afolayan A.J. (2019). Helminthiases; prevalence, transmission, host-parasite interactions, resistance to common synthetic drugs and treatment. Heliyon.

[j_helm-2025-0012_ref_017] Jemâa J.B., Tersim N., Toudert K.T., Khouja M.L. (2012). Insecticidal activities of essential oils from leaves of *Laurus nobilis* L. from Tunisia, Algeria and Morocco, and comparative chemical composition. J Stored Prod Res.

[j_helm-2025-0012_ref_018] Weinstock J.V., Elliott D.E. (2009). Helminths and the IBD hygiene hypothesis. Inflamm Bowel Dis.

[j_helm-2025-0012_ref_019] Kasl E.L., Font W.F., Criscione C.D. (2018). Resolving evolutionary changes in parasite life cycle complexity: Molecular phylogeny of the trematode genus *Alloglossidium* indicates more than one origin of precociousness. Mol Phylogenet Evol.

[j_helm-2025-0012_ref_020] Kivçak B, Mert T. (2002). Preliminary evaluation of cytotoxic properties of *Laurus nobilis* leaf extracts. Fitoterapia.

[j_helm-2025-0012_ref_021] Koike A., Barreira J.C., Barros L., Santos-Buelga C., Villavicencio A.L., Ferreira I.C. (2015). Edible flowers of *Viola tricolor* L. as a new functional food: Antioxidant activity, individual phenolics and effects of gamma and electron-beam irradiation. Food Chem.

[j_helm-2025-0012_ref_022] Manikandan P., Letchoumy P.V., Gopalakrishnan M., Nagini S. (2008). Evaluation of *Azadirachta indica* leaf fractions for *in vitro* antioxidant potential and *in vivo* modulation of biomarkers of chemoprevention in the hamster buccal pouch carcinogenesis model. Food Chem Toxicol.

[j_helm-2025-0012_ref_023] Matte E.C., Luciano F.B., Evangelista A.G. (2023). Essential oils and essential oil compounds in animal production as antimicrobials and anthelmintics: an updated review. Anim Health Res Rev.

[j_helm-2025-0012_ref_024] Merghni A., Marzouki H., Hentati H., Aouni M., Mastouri M. (2016). Antibacterial and antibiofilm activities of *Laurus nobilis* L. essential oil against *Staphylococcus aureus* strains associated with oral infections. Curr Res Transl Med.

[j_helm-2025-0012_ref_025] Nalule A.S., Mbaria J.M., Kimenju J.W. (2013). *In vitro* anthelmintic potential and phytochemical composition of ethanolic and water crude extracts of *Euphorbia heterophylla* Linn. J. Med. Plants Res.

[j_helm-2025-0012_ref_026] Oliveira L.B., Bevilaqua C.M.L., Costa C.C., Macedo I.F., Barros R.S., Rodrigues A.M., Navarro A.C. (2009). Anthelmintic activity of *Cocos nucifera* L. against sheep gastrointestinal nematodes. Vet Parasitol.

[j_helm-2025-0012_ref_027] Omer S.A., Alghamdi J.M., Alrajeh A.H., Aldamigh M., Mohammed O.B. (2020). Morphological and molecular characterization of *Aspiculuris tetraptera* (nematoda: Heteroxynematidae) from *Mus musculus* (rodentia: Muridae) in Saudi Arabia. Biosci Rep.

[j_helm-2025-0012_ref_028] Paparella A., Nawade B., Shaltiel-Harpaz L., Ibdah M. (2022). A review of the botany, volatile composition, biochemical and molecular aspects, and traditional uses of *Laurus nobilis*. Plants.

[j_helm-2025-0012_ref_029] Ranasinghe S., Armson A., Lymbery A.J., Zahedi A., Ash A. (2023). Medicinal plants as a source of antiparasitics: an overview of experimental studies. Pathog Glob Health.

[j_helm-2025-0012_ref_030] Sayed E., Altilmisani N.M., Albishri F., Ahmed A., Elkhalifa S.M., Al-Dubai T.A., Al-Wesabi E.O. (2024). Prevalence and zoonotic potential of parasites in wild rats in Jeddah City, Saudi Arabia. Int J Vet Sci.

[j_helm-2025-0012_ref_031] Scantlebury M., Waterman J.M., Hillegass M., Speakman J.R., Bennett N.C. (2007). Energetic costs of parasitism in the Cape ground squirrel *Xerus inauris*. Proc Biol Sci.

[j_helm-2025-0012_ref_032] Sebai E., Abidi A., Benyedem H., Dhibi M., Hammemi I., Akkari H. (2022). Phytochemical profile and anthelmintic effects of *Laurus nobilis* essential oil against the ovine nematode *Haemonchus contortus* and the murine helminth model *Heligmosomoides polygyrus*. Vet Parasitol.

[j_helm-2025-0012_ref_033] Simić A., Soković M.D., Ristić M., Grujić-JovAnović S., vukojević J., Marin P.D. (2004). The chemical composition of some Lauraceae essential oils and their antifungal activities. Phytother Res.

[j_helm-2025-0012_ref_034] Siriken B., Yavuz C., Guler A. (2018). Antibacterial Activity of *Laurus nobilis*: A review of literature. Med. Sci. Discov..

[j_helm-2025-0012_ref_035] Stepek G., Buttle D.J., Duce I.R., Lowe A., Behnke J.M. (2005). Assessment of the anthelmintic effect of natural plant cysteine proteinases against the gastrointestinal nematode, *Heligmosomoides polygyrus, in vitro*. Parasitology.

[j_helm-2025-0012_ref_036] Stepek G., Lowe A.E., Buttle D.J., Duce I.R., Behnke J.M. (2006). *In vitro* and *in vivo* anthelmintic efficacy of plant cysteine proteinases against the rodent gastrointestinal nematode, *Trichuris muris*. Parasitology.

[j_helm-2025-0012_ref_037] Mares M.M., Murshed M., Aljawdah H., Al-Quraishy S. (2024). The assessment of the anthelmintic activity of *Laurus nobilis* extract in mice naturally infected with *Aspiculuris tetraptera*. Indian J Anim Res.

[j_helm-2025-0012_ref_038] Yang F., Long E., Wen J., Cao L., Zhu C., Hu H., Lv Z. (2014). Linalool, derived from *Cinnamomum camphora* (L.) Presl leaf extracts, possesses molluscicidal activity against Oncomelania hupensis and inhibits infection of *Schistosoma japonicum*. Parasit Vectors.

[j_helm-2025-0012_ref_039] Verdú J.R., Cortez V., Rosa-Garcia R., Ortiz A.J., García-Prieto U., Lumaret J.P., Sánchez-Pinero F. (2023). Nontoxic effects of thymol, carvacrol, cinnamaldehyde, and garlic oil on dung beetles: A potential alternative to ecotoxic anthelmintics. PLoS One.

[j_helm-2025-0012_ref_040] Williams A.R., Ramsay A., Hansen T.V., Ropiak H.M., Mejer H., Nejsum P., Mueller-Harvey I., Thamsborg S.M. (2015). Anthelmintic activity of trans-cinnamaldehyde and A- and B-type proanthocyanidins derived from cinnamon (*Cinnamomum verum*). Sci Rep.

